# Die Covid-19-Pandemie: Schlaglicht auf das Spannungsfeld von Biowaffenkontrolle, Biosicherheit und globaler Gesundheit

**DOI:** 10.1007/s42597-020-00039-9

**Published:** 2020-10-07

**Authors:** Una Jakob

**Affiliations:** Leibniz-Institut Hessische Stiftung Friedens- und Konfliktforschung (HSFK), Frankfurt/Main, Baseler Straße 27–31, 60329 Frankfurt am Main, Deutschland

**Keywords:** Biowaffen, Biosicherheit, Globale Gesundheit, Pandemie, Abrüstung, Biological weapons, Biosecurity, Global health, Pandemic, Disarmament

## Abstract

Die Covid19-Pandemie ist höchstwahrscheinlich auf einen natürlichen Krankheitsausbruch zurückzuführen. Trotzdem können Anschuldigungen, sie sei durch die absichtliche oder fahrlässige Freisetzung des neuartigen Corona-Virus herbeigeführt worden, bestehende Konflikte und Rivalitäten verstärken. Besitz und Einsatz biologischer Waffen sind völkerrechtlich verboten. Mangelnde Transparenz, fehlende Kontrollen und legitime biologische Forschungen mit hohem Missbrauchspotenzial lassen aber Raum für Desinformation, Fehlwahrnehmungen und Verdächtigungen, wie sie auch am Beispiel der Covid19-Pandemie zu finden sind. Bessere Verifikations- und Untersuchungsmöglichkeiten für das Biowaffenverbot, klarere Regeln für den Umgang mit Verstößen dagegen sowie größere Transparenz im Bereich der sicherheitsrelevanten biologischen Forschung könnten helfen, mögliche Biowaffeneinsätze zu verhindern und negative politische Dynamiken bei ungewöhnlichen Krankheitsausbrüchen einzuhegen. Hierfür müsste unter anderem das Biowaffen-Übereinkommen gestärkt und das Zusammenspiel von biologischer Abrüstung, Biosicherheit und globaler Gesundheit deutlicher fokussiert werden. Eine klare Arbeitsteilung und die Nutzung von Synergien, z. B. in der Gesundheitsvorsorge und im Umgang mit wissenschaftlichen und technologischen Entwicklungen, kämen sowohl der Pandemievorsorge als auch der biologischen Abrüstung zugute.

## Abstract (en)

The Covid19 pandemic can most likely be traced to a natural outbreak. Nevertheless, allegations that the novel corona virus was intentionally or accidentally released from a laboratory could exacerbate existing conflicts and tensions. The possession and use of biological weapons are prohibited by international law. The lack of transparency and of controls as well as legitimate biological research with high potential for misuse leave room for misinformation, misperceptions and suspicions, as has been the case with the Covid19 pandemic. Stronger mechanisms for verifying and investigating the bioweapons prohibition, stricter compliance procedures to deal with violations, and greater transparency for dual-use research of concern could help prevent biological attacks and contain negative political dynamics in case of unusual disease outbreaks. To that end, the Biological Weapons Convention would need to be strengthened, and the interplay of biological disarmament, biosecurity and global health would need to be more focussed. Having a clear division of labour and exploiting synergies, e.g. in health preparedness and in dealing with scientific and technological developments, would benefit both pandemic preparedness and biological disarmament.

## Einleitung: Die Covid19-Pandemie und das Spektrum biologischer Risiken

Berichte von Pandemien sind seit der Antike überliefert (World Economic Forum 2020). Im letzten Jahrhundert gab es gleich mehrere solcher globalen Ausbrüche von Infektionskrankheiten, wobei der verheerendste die „Spanische Grippe“ war, die zwischen 1918 und 1920 weltweit geschätzte 20–50 Mio. Opfer forderte (WHO 2017, S. 26). Von solchen Dimensionen ist die Covid19-Pandemie derzeit weit entfernt. Dennoch dürfte sie mit ihrer Ausbreitung in praktisch alle Länder und Territorien der Erde sowie hinsichtlich der globalen wirtschaftlichen, politischen und sozialen Folgen neue Maßstäbe setzen.

Höchstwahrscheinlich ist diese Pandemie auf einen natürlichen Krankheitsausbruch zurückzuführen (s. Andersen et al. 2020). Theoretisch können jedoch verschiedene Ereignisse Infektionskrankheiten ausbrechen lassen. Das Spektrum biologischer Risiken reicht dabei vom Einsatz biologischer Waffen, bioterroristischen oder kriminellen Aktionen über versehentliche Freisetzungen aus Forschungslaboratorien zu natürlichen Krankheitsausbrüchen. Die gesundheitlichen Folgen unterscheiden sich bei gleichen Erregern dabei nicht zwangsläufig. Die politischen Dynamiken und Konsequenzen können dagegen ganz unterschiedliche Formen annehmen. Die Frage nach dem Ursprung einer Pandemie ist daher nicht nur epidemiologisch, sondern auch politisch bedeutsam.

In der Covid19-Pandemie wurden diverse Szenarien bezüglich ihres Ursprungs in Umlauf gebracht. So wurden in der Frühphase Verdächtigungen laut, es habe sich um einen absichtlichen Angriff – wahlweise Chinas oder der USA – gehandelt, oder das Virus entstamme einem geheimen Biowaffen-Forschungsprogramm (s. z. B. Field und Krzyzaniak 2020). Auch die Freisetzung durch nicht-staatliche Akteure – etwa Bill Gates – kursiert in Verschwörungstheorien (Lynas 2020a), und der Einsatz von SARS-CoV‑2 oder vergleichbaren Erregern durch terroristische Organisationen wird als hypothetische oder zukünftige Option diskutiert, unter anderem von VN-Generalsekretär Gueterres (Gueterres 2020; s. auch z. B. Mullins 2020). Während sich die Anschuldigungen vor allem im politischen Diskurs finden, stehen ihnen naturwissenschaftliche Erkenntnisse gegenüber, die kaum Zweifel an einem natürlichen Ausbruch lassen (Andersen et al. 2020; Calisher et al. 2020; Scheid und Zöller 2020). Nach wie vor steht auch der Vorwurf im Raum, SARS-CoV‑2 sei aus einem zivilen chinesischen Forschungslabor entwichen (s. z. B. Field 2020; Lentzos 2020; Leitenberg 2020).

Die politischen Diskussionen um Verantwortlichkeiten im Zusammenhang mit der Pandemie spiegeln erstens den aktuellen Stand der Weltpolitik, insbesondere die Rivalitäten zwischen den USA, China und Russland; die Pandemie bietet hier eine weitere Gelegenheit, die politischen Gegner zu diskreditieren, vielleicht auch, um vom eigenen Missmanagement der Krise abzulenken (s. Horsley 2020). Wie schnell dies zum Stolperstein in den internationalen Beziehungen werden kann, illustriert die Anekdote, dass sich die Außenminister der G7-Staaten im März 2020 nicht auf eine gemeinsame Stellungnahme zur Pandemie einigen konnten, weil die USA auf der Bezeichnung „Wuhan-Virus“ für SARS-CoV‑2 bestanden (Tagesschau 2020) Zweitens werfen die Diskussionen ein Schlaglicht auf Probleme in einem konkreten Politikfeld: der Kontrolle biologischer Waffen und der biologischen Sicherheit an der Schnittstelle zur globalen Gesundheit (s. auch Jakob 2020). Sie zeigen, wie schnell der Verdacht aufkommen (oder aufgebracht werden) kann, ein Krankheitsausbruch sei absichtlich oder fahrlässig herbeigeführt worden, und wie schwierig es mit den vorhandenen Mitteln ist, mit einem solchen Verdacht konstruktiv umzugehen und die Folgen einzuhegen. Der Verdacht auf fahrlässige Freisetzung kann etwa Regressforderungen nach sich ziehen und den Ruf einer Forschungseinrichtung oder Regierung schädigen. Auf vermutete nicht-staatliche Aktionen können Antiterrormaßnahmen folgen, die je nach ihrer Verhältnismäßigkeit politische und gesellschaftliche Spannungen verursachen könnten. Schließlich kann der Vorwurf eines illegalen Biowaffenprogramms und -einsatzes bestehende internationale Konflikte verschärfen, neue entstehen lassen und Rüstungsdynamiken in Gang setzen, die schlimmstenfalls in einem biologischen Wettrüsten münden könnten.

Die Einhegung solcher Dynamiken zählt zu den Aufgaben der Rüstungskontrolle, und mit dem Biowaffen-Übereinkommen (BWÜ) von 1972 steht ein internationaler Vertrag bereit, der die biologische Abrüstung dauerhaft gewährleisten soll. Allerdings fehlen ihm derzeit die Möglichkeiten, den oben beschriebenen Entwicklungen wirksam zu begegnen. Dieser Beitrag konzentriert sich darauf, diese Dimension der „Corona-Krise“ zu beleuchten und Handlungsoptionen zu diskutieren. Hierfür wird zunächst das BWÜ-Regime vorgestellt und es wird konkret ausgeführt, wie eine biologische Rüstungsdynamik in Gang gesetzt und verhindert werden könnte. Verifikation, ein Untersuchungsmechanismus für ungewöhnliche Krankheitsausbrüche sowie größere Transparenz im Bereich militärischer und ziviler sicherheitsrelevanter Forschung werden als diejenigen Bereiche präsentiert, in denen Fortschritte nötig sind, um das BWÜ-Regime an diese aktuelle Herausforderung anzupassen. Abschließend wird aufgezeigt, welche ersten Schritte in diese Richtung auch in der gegenwärtig angespannten internationalen Lage möglich sein könnten.

## Das BWÜ-Regime: Stand der Dinge und Implikationen für ungewöhnliche Krankheitsausbrüche

Unter biologischen Waffen versteht man Mikroorganismen und Toxine^4^ sowie geeignete Ausbringungsmittel, die zusammen mit der Absicht verwendet werden, Menschen, Tiere oder Pflanzen krankzumachen oder zu töten. Nicht alle Krankheitserreger eignen sich dafür gleichermaßen; für die Beurteilung der Nützlichkeit als Biowaffe ist je nach militärischem Einsatzszenario unter anderem entscheidend, ob die Erreger von Mensch zu Mensch übertragbar sind, wie leicht sie sich verbreiten und wie schwerwiegend ihre Folgen sind, wie empfindlich sie auf Umwelteinflüsse reagieren und ob Schutzmaßnahmen und Gegenmittel zur Verfügung stehen.

Das neuartige Corona-Virus SARS-CoV‑2 ist keine geeignete Biowaffe: Es breitet sich schnell und unkontrolliert aus, und potenzielle Angreifende haben keine Möglichkeit, die eigenen Truppen oder Bevölkerungen davor zu schützen. Militärisch ist es damit nutzlos. Selbst für terroristische Gruppen würde ein Angriff nur Sinn ergeben, wenn die Organisation keinerlei Rücksicht auf eine eigene Unterstützungs- und Rekrutierungsbasis nehmen müsste (s. Jakob 2020). Zudem haben genetische Untersuchungen keine Hinweise darauf ergeben, dass das Virus in irgendeiner Form gentechnisch manipuliert worden wäre (s. Lynas 2020b). Ein Biowaffenangriff oder terroristischer Anschlag mit SARS-CoV‑2 erscheint damit zum jetzigen Zeitpunkt äußerst unwahrscheinlich. Dazu passt, dass die entsprechenden Anschuldigungen nur von einzelnen Akteuren stammten und sich nicht lange hielten. Sie fügten sich allerdings nahtlos in breitere, bestehende politische Rivalitäten und Spannungen ein: So beschuldigten sich vor allem die USA und China gegenseitig, und auch von iranischer Seite wurden Anschuldigungen gegen die USA laut, während die EU Russland vorwarf, eine Desinformationskampagne zu steuern (Aljazeera 2020; Deuber 2020; Emmott 2020; von Hein 2020; Holland 2020).

Der Einsatz biologischer Waffen im Krieg ist seit 1925 durch das Genfer Protokoll und mittlerweile völkergewohnheitsrechtlich verboten (Henckaerts und Doswald-Beck 2005). Seit 1975 verbietet das Biowaffen-Übereinkommen (BWÜ) zudem seinen aktuell 183 Mitgliedern Besitz, Herstellung und Weitergabe solcher Waffen. Zu den zentralen Bestimmungen des BWÜ zählen neben dem umfassenden Biowaffenverbot (Artikel I) unter anderem die Verpflichtung zur biologischen Abrüstung und Nichtverbreitung (Artikel II und III), zur Umsetzung der Vertragsbestimmung in nationales Recht (Artikel IV), zur Hilfeleistung im Falle eines Biowaffenangriffs (Artikel VII) sowie zur internationalen Kooperation bei der friedlichen Nutzung der Biologie und Biotechnologie (Artikel X). Im Falle von Problemen bei der Anwendung der Konvention können die Mitglieder untereinander konsultieren (Artikel V) oder bei Vertragsverstößen den VN-Sicherheitsrat anrufen (Artikel VI). Alle fünf Jahre werden Überprüfungskonferenzen abgehalten, bei denen sich die Vertragsstaaten über die Wirkweise und Auslegung des Vertrags verständigen und bei denen politisch bindende Beschlüsse über ergänzende Maßnahmen vereinbart werden können. So wurden beispielsweise in den 1980er Jahren Konsultationsprozeduren ausgearbeitet und Vertrauensbildende Maßnahmen (VBM) beschlossen. Seit 2002 finden zwischen den Überprüfungskonferenzen jährliche Experten- und Vertragsstaatentreffen statt, deren Agenden jeweils auch von den Überprüfungskonferenzen ausgehandelt und festgelegt werden. 2006 wurde eine kleine *Implementation Support Unit (ISU)* eingerichtet, die die Interaktion der Vertragsstaaten unterstützt. Während das Biowaffenverbot nicht in Frage steht, krankt das zugehörige Regime an mehreren Defiziten, u. a. im Bereich der Verifikation und Institutionalisierung. Tiefe politische Gegensätze behindern eine Weiterentwicklung und Stärkung seit Jahren.^6^

Der Vorwurf, ein Staat betreibe ein geheimes Biowaffenprogramm, wiegt schwer, handelt es sich doch um geächtete Waffen, deren Einsatz und Verbreitung der VN-Sicherheitsrat als Gefahr für den Weltfrieden und die internationale Sicherheit postuliert hat (s. z. B. Resolution 1540 (2004)). Geben ungewöhnliche Krankheitsausbrüche Anlass zu solchen Vermutungen, oder werden sie für politische motivierte Anschuldigungen ausgenutzt, können sich dadurch, wie oben beschrieben, schwer zu kontrollierende Konfliktdynamiken ergeben. So können etwa legitime defensive Forschungsaktivitäten in ihrem Umfang überschätzt oder als offensiv fehlgedeutet werden, was wiederum intensivierte Bioabwehrforschung oder gar offensive Planungen provozieren kann. Da es im biologischen Bereich schwierig ist, zwischen zivilen, defensiven und offensiven Forschungen zu unterscheiden (Dual-Use-Problematik), besteht bei mangelnder Transparenz das Risiko, dass Fehlwahrnehmungen oder falsche Anschuldigungen in eine biologische Rüstungsspirale münden und/oder bestehende politische Spannungen verschärfen. Um solchen Dynamiken vorzubeugen oder wirksam zu begegnen, müssten vor allem zwei Bereiche der Biowaffenkontrolle gestärkt werden: die Möglichkeiten, schon vor einem Verdachtsfall die Vertragstreue der BWÜ-Mitglieder zu überprüfen sowie die Chancen, den Ursprung eines verdächtigen Krankheitsausbruchs unabhängig zu untersuchen.

## Verifikation des BWÜ und die Untersuchung von ungewöhnlichen Krankheitsausbrüchen als Maßnahmen der Krisenprävention

Das BWÜ enthält keinerlei Verifikationsmaßnahmen. Dadurch konnte weder die Abrüstung früherer offensiver Biowaffen-Programm unabhängig überprüft und dokumentiert werden, noch gibt es aktuell Möglichkeiten, allen Mitgliedern die nachweisliche Vertragseinhaltung zu attestieren. Durch die oben beschriebene Dual-Use-Problematik ist auch die Verifikation des Biowaffenverbots besonders anspruchsvoll (Koblentz 2009, S. 64–74) und bleibt viel Raum für Missverständnisse, Vermutungen und Verdächtigungen. So stellen die USA seit Jahren infrage, ob China und Russland ihre früheren Biowaffenaktivitäten vollständig eingestellt haben (z. B. US DoS 2020). Umgekehrt wurden die USA wiederholt für Bioabwehrforschungen kritisiert, die sich am Rande der Legalität bewegten und offensive Optionen offenließen (z. B. Reeves et al. 2018; Wheelis und Dando 2003). Russland unterstellt Washington zudem, in Labors in anderen Ländern verbotene offensive Forschungen durchzuführen (Lentzos 2018). Die rasanten wissenschaftlichen und technologischen Entwicklungen in der Biologie, Biotechnologie und anderen Disziplinen – zum Beispiel in der Gentechnologie, synthetischen Biologie oder im Bereich der künstlichen Intelligenz – vergrößern diesen Spielraum. Sie wecken außerdem Befürchtungen, dass biologische Waffen zukünftig militärisch interessanter und leichter zu beschaffen sein könnten, als es bisher der Fall ist (s. z. B. Brockmann et al. 2019; Kelle et al. 2012; Nixdorff 2018; Spiez 2018).

Das Verifikationsdefizit wurde im BWÜ-Regime nicht mehr konstruktiv bearbeitet, seit im Jahr 2001 die Verhandlungen über ein Compliance-Protokoll scheiterten (Littlewood 2005). Stattdessen rückten Maßnahmen in den Fokus, die die nationale Implementierung des BWÜ sowie die Bereitschaft der Mitgliedsstaaten zur Abwehr biologischer Gefahren des gesamten Spektrums stärken sollten. Gesteigerte Bedeutung kam auch der Frage zu, wie mit den wissenschaftlichen und technologischen Fortschritten umgegangen werden kann, um mögliche Proliferationsrisiken und Missbrauchsmöglichkeiten frühzeitig zu erkennen und zu minimieren, gleichzeitig aber auch ihre Chancen weitestmöglich nutzen zu können. Fragen der biologischen Sicherheit, also des Schutzes vor versehentlichem oder absichtlichem Freisetzen von Erregern aus Laboratorien^7^, sind zudem seit 2001 zwar nicht unumstrittene, aber inzwischen etablierte Bestandteile der Diskussionen im Regime.

Ein gut funktionierendes Gesundheitssystem stellt ein zentrales Element dar, um die Folgen von Krankheitsausbrüchen jeglicher Ursache abzufedern und um biologische Angriffe von Staaten oder nicht-staatlichen Akteuren abzuschrecken. Mit der Hinwendung zu nationalen Implementierungsmaßnahmen, Terrorismusprävention, *preparedness* und der von der Gruppe der blockfreien Staaten (*Non-Aligned Movement*, NAM) durchgesetzten stärkeren Berücksichtigung der Entwicklungsdimension in der friedlichen Nutzung der Biotechnologie gewann die nationale und globale öffentliche Gesundheit, einschließlich der Pandemievorsorge, innerhalb des BWÜ-Regimes stärker an Bedeutung. Klassische Abrüstungselemente des BWÜ wie die Verifikationsfrage traten demgegenüber in den Hintergrund.

Nur mit einem wirksamen Verifikations- und Compliancesystem ließe sich jedoch das Vertrauen in die Vertragstreue der Mitglieder erhöhen und könnten Beschuldigte unberechtigte Vorwürfe glaubhafter zurückweisen. Mit der qualitativen Entwicklung und geographischen Verbreitung des Biotechnologie-Sektors hat sich auch die Art und Zahl der relevanten Akteure vervielfacht. Ein zeitgemäßes Verifikationssystem müsste also einen Mix aus klassischen Verifikationselementen und Governance-Strategien enthalten, die dem aktuellen Multi-Stakeholder-Setting gerecht werden (s. auch Lennane 2011; Lentzos 2013; Revill 2017), und es müsste sowohl auf der Ebene staatlicher Aktivitäten als auch bei der Sicherung gefährlicher Erreger ansetzen. Deklarationen, Transparenzmaßnahmen und Inspektionen („Besuche“), wie sie für das Compliance-Protokoll vorgesehen waren, könnten etwa kombiniert werden mit Aufklärungsinitiativen und Verhaltenskodizes für Forschende, an sicherheitswahrende Auflagen gebundene Forschungsförderung und globale, verbindliche Standards für den Umgang mit gefährlichen Krankheitserregern.

Ein solches System böte zwar keine absolute Gewissheit; über die genauere Kenntnis von üblichen Aktionsmustern und Abläufen erhöhte sich aber die Entdeckungswahrscheinlichkeit für Unregelmäßigkeiten und damit das Vertrauen in die Funktionsfähigkeit des BWÜ-Regimes und in die Vertragstreue seiner Mitglieder. Die beschriebenen Risiken durch Fehlwahrnehmungen oder gezielte Desinformation ließen sich verringern. Inhaltlich spiegelte ein solches System die Verschmelzung von Aspekten der biologischen Abrüstung, Biosicherheit und globalen Gesundheit, die sich in den letzten Jahren im BWÜ-Regime beobachten lässt.

Verifikationsmaßnahmen stellen ein wichtiges stabilisierendes Element dar; im Falle eines ungewöhnlichen Krankheitsausbruchs ist daneben aber auch die Untersuchung seines Ursprungs essenziell. Auch eine solche Untersuchung sollte den beschriebenen Entwicklungen in der Biowaffenkontrolle Rechnung tragen und das gesamte Spektrum biologischer Risiken abdecken können. Für die Untersuchung von Krankheitsausbrüchen ist auf internationaler Ebene primär die Weltgesundheitsorganisation (WHO) zuständig. Sie führt entsprechende Untersuchungen auf epidemiologischer Ebene durch; hier gilt es unter anderem, die ersten Infektionsorte, die ersten Patienten und Patientinnen und eventuelle Wirte zu identifizieren sowie Übertragungswege zu erforschen. Eine Untersuchung der Covid-19-Pandemie und ihrer Quellen war im August 2020 in Vorbereitung; eine erste Sondierungs- und Vorbereitungsmission wurde im Juli 2020 durchgeführt (WHA 2020).^2220^ Für die Tatsachenermittlung jenseits epidemiologischer Fragen hat die WHO allerdings kein Mandat.

Gäbe es einen soliden Verdacht auf einen staatlichen Biowaffeneinsatz, stünde für die Untersuchung ein Mechanismus des VN-Generalsekretärs (UNSGM) bereit. Der UNSGM wurde in den 1980er Jahren aufgebaut und kann bei vermuteten Bio- oder Chemiewaffeneinsätzen aktiviert werden.^9^ Bei Covid19 gäbe es dafür zu wenige belastbare Hinweise. Da aber schon bloße Anschuldigungen in der ohnehin angespannten Pandemie-Situation für eine Konflikteskalation sorgen könnten, wäre es grundsätzlich hilfreich, wenn ein internationaler Mechanismus die Tatsachenermittlung bei ungewöhnlichen Krankheitsausbrüchen mit unklarer Ursache übernehmen könnte, der alle möglichen Quellen eines Ausbruchs gleichermaßen in den Blick nehmen kann. Der UNSGM könnte hier eine nützliche Funktion übernehmen, entweder als Modell für ein neu zu schaffendes Instrument oder als Gerüst, das für diesen erweiterten Zweck ausgebaut werden könnte. Voraussetzung für letzteres wäre eine Mandatsänderung per Resolution der VN-Generalversammlung oder des VN-Sicherheitsrats. Untersuchungen ungewöhnlicher Krankheitsausbrüche könnten Vorwürfe der absichtlichen Ausbringung oder fahrlässigen Freisetzung, wie sie aktuell vor allem gegen China geäußert werden, aufklären helfen und eine sachliche Grundlage für die Formulierung von politischen Reaktionen auf einen Ausbruch liefern.^10^

Um zu verhindern, dass künftige Krankheitsausbrüche von überregionaler Reichweite – durch Instrumentalisierung, Fehlwahrnehmungen oder ambivalente Informationslage – biologische Rüstungsdynamiken auslösen oder sicherheitspolitische Krisen verstärken, und um einen absichtlichen Biowaffeneinsatz weitestmöglich zu verhüten, müsste das BWÜ-Regime also hinsichtlich der Verifikation und der Untersuchungsmöglichkeiten von ungewöhnlichen Krankheitsausbrüchen unterstützt und gestärkt werden (s. auch Lentzos 2019; Revill 2017). Die aktuelle politische Lage im BWÜ-Regime und im VN-Sicherheitsrat macht neue rechtlich verbindliche Maßnahmen in naher Zukunft allerdings unwahrscheinlich. Politisch verbindliche oder freiwillige Transparenzmaßnahmen, die über das BWÜ hinausreichen, könnten zumindest eine Zwischenlösung bereitstellen.

## Transparenz und Vertrauensbildung an der Schnittstelle von Abrüstung, Biosicherheit und globaler Gesundheit

Die rüstungskontrollpolitische Strategie, über Vertrauensbildende Maßnahmen (VBM) die Transparenz zu steigern, wird im BWÜ-Regime bereits seit den 1980er Jahren verfolgt. 1986 und 1991 wurden die noch heute gültigen politisch verbindlichen VBM eingeführt, und 2011 wurden sie leicht überarbeitet (Becker-Jakob 2013, S. 27–29). Drei der VBM-Formulare fragen Informationen über ungewöhnliche Krankheitsausbrüche, frühere offensive Biowaffenprogramme sowie Aktivitäten in der Bioabwehrforschung ab.^11^ So könnten sie bei optimaler Nutzung hilfreiche Informationen bereitstellen, um in Fällen wie der Covid19-Pandemie Ungewissheit zu reduzieren und falsche Verdächtigungen zu erschweren. Vorwürfe wie die, China und Russland hätten frühere offensive Aktivitäten nie vollständig eingestellt, oder die USA betrieben illegale Offensivforschung in Einrichtungen außerhalb des eigenen Landes (s. oben), ließen sich bei größtmöglicher Transparenz überzeugender vorbringen oder zurückweisen. Allerdings beteiligt sich nur ein Teil der BWÜ-Vertragsstaaten regelmäßig oder überhaupt am VBM-Austausch, und dieser beschränkt sich auf die bloße Abgabe der Informationen ohne weitere kollektive Aufarbeitung oder Überprüfung der übermittelten Daten. Reformen wären deshalb dringend nötig.

Einen anderen Ansatzpunkt bieten innerhalb des Regimes die seit einigen Jahren praktizierten „Peer Reviews“ (s. Revill 2013). Dieses von Frankreich in den BWÜ-Diskurs eingebrachte Konzept sah ursprünglich vor, dass Staaten auf freiwilliger und kooperativer Basis ihre nationalen BWÜ-Implementierungsmaßnahmen interessierten Staaten zur Begutachtung freigeben, um sich Verbesserungsmöglichkeiten aufzeigen zu lassen und die eigene Vertragstreue zu demonstrieren. Erweitert um eine Transparenzkomponente in der Bioabwehrforschung wurden mittlerweile auch „peer review transparency visits“ in staatlichen Forschungslabors veranstaltet (Deutschland 2016; Georgien 2018). Georgien konnte so russischen Vorwürfen vertragswidriger Forschungen begegnen und Beobachterinnen und Beobachter von der vertragskonformen Natur der Aktivitäten in der besuchten georgischen Anlage überzeugen (Lentzos 2018). China stünde es natürlich frei, in einem ähnlichen Prozess die eigene einschlägige Gesetzgebung offenzulegen und über Biosicherheitsmaßnahmen, einschließlich jener in den nahe am vermuteten Ursprungsort der Pandemie gelegenen Forschungseinrichtungen, zu informieren. Wie viele blockfreie Staaten und Russland lehnt aber auch China das Peer Review-Konzept im BWÜ ab und fordert stattdessen eine Rückkehr zu den Protokollverhandlungen (z. B. China 2016).

Bestehende Transparenzmaßnahmen innerhalb des BWÜ verfolgen also hilfreiche Absichten, stoßen aber aus mehreren Gründen an Grenzen. Zum einen sind sie abhängig von der Qualität und Quantität der übermittelten Informationen und von ihrer politischen Akzeptanz. Zum anderen sind sie staatenzentriert, bilden damit nicht mehr die Realität der Forschungslandschaft ab und werden weder dem Tempo der biotechnologischen Entwicklung noch den Überschneidungen zwischen biologischer Abrüstung, Biosicherheit und globaler Gesundheit gerecht. Die Problematik lässt sich besonders gut am Beispiel sicherheitsrelevanter Forschung illustrieren. Darunter versteht man Forschungen und Experimente, die an sich legitim und nützlich sind, aber ein besonders hohes Missbrauchs- und Risikopotenzial bergen (*dual-use reserach of concern*, DURC). Beispiele dafür finden sich auch in der Pandemieprävention, die im Bereich der globalen Gesundheitsvorsorge und Bekämpfung von Infektionskrankheiten einen wichtigen Platz einnimmt. Auch in diesem Bereich wurden sogenannte „gain of function“-Forschungen durchgeführt, bei denen zum besseren Verständnis von Übertragungswegen, Immunreaktionen oder möglichen Mutationen Erreger gentechnisch so manipuliert werden, dass ihre Pathogenität steigt. In vorbeugender Absicht verstärken solche Experimente also möglicherweise einige der Risiken, die sie eigentlich bekämpfen sollen (s. Casadevall und Imperiale 2014). Größere Transparenz, einheitliche Richtlinien und standardisierte Sicherungsmaßnahmen könnten hier Risiken minimieren helfen. Den sicheren Umgang mit solchen Forschungen zu gewährleisten, ohne dabei medizinischen Fortschritt zu bremsen und die Freiheit der Wissenschaft ungebührlich zu beschränken, stellt aktuell eine besondere Herausforderung dar.

## Fazit

Die Covid19-Pandemie hat aufgezeigt, welche Chancen und Probleme das BWÜ-Regime im Umgang mit einer solchen Ausnahmesituation hat. Auch bei einem natürlichen Krankheitsausbruch besteht das Risiko, dass durch absichtliche oder unabsichtliche Anschuldigungen oder durch Fehleinschätzungen einer Lage negative politische (Rüstungs‑)Dynamiken in Gang gesetzt werden. Die Reduktion von Unsicherheiten und Fehlwahrnehmungen, um Eskalationen und Rüstungsspiralen zu verhüten, zählt zu den klassischen Zielen der Rüstungskontrolle. Die Pandemie zeigt, dass dieses Ziel im Kern heute noch relevant ist – vielleicht sogar relevanter denn je angesichts technologischer Fortschritte und der Erwartungswahrscheinlichkeit für größere Ausbruchsgeschehen. Um diesen Dynamiken und Entwicklungen etwas entgegensetzen zu können, müsste das BWÜ allerdings an aktuelle Erfordernisse angepasst werden.

Insbesondere im Umgang mit DURC verschwimmen die Grenzen zwischen klassischer biologischer Abrüstung und Biosicherheit, da es nicht länger nur darum geht, staatliche Proliferation zu verhindern, sondern Risiken des gesamten Spektrums zu minimieren. Transparenzmaßnahmen sind hierbei ein zentrales Element; sie sollten nicht nur staatliche Bioabwehr- und andere Aktivitäten abdecken, sondern zum Beispiel auch sicherheitsrelevante Forschung wie DURC-Experimente. Aufgrund der ebenfalls betroffenen substaatlichen Sektoren und Ebenen – Wissenschaft, Privatwirtschaft bis hin zur Rolle einzelner Forschenden – gerät das BWÜ als Steuerungsinstrument jedoch an seine Grenzen. Es ist nicht das geeignete Forum dafür, alle biologischen Risiken gleichermaßen zu bearbeiten, kann aber helfen, die (bio-)politischen „Nebenwirkungen“ von Epidemien und Pandemien abzumildern. Dafür müssten die Schnittmengen des BWÜ mit Biosicherheits- und Gesundheitsthemen noch stärker als bisher in die Diskussion von Handlungsoptionen einbezogen werden, z. B. in einer möglichen Überarbeitung der VBM oder in zukünftigen Diskussionen um Verifikationsmöglichkeiten. Eine Chance dafür böte sich theoretisch bereits 2021 im Rahmen der 9. BWÜ-Überprüfungskonferenz.

Gleichzeitig sollten die unterschiedlichen Mandate und Zielsetzungen aller Bereiche explizit gemacht und Zuständigkeiten klar abgegrenzt werden. So sollte die Pandemieprävention und Vorsorge (*preparedness*) im Zuständigkeitsbereich der WHO verbleiben. Das BWÜ sollte sich auf seine Kernaufgaben konzentrieren und die biologische Abrüstung sicherstellen sowie biologischen Rüstungsdynamiken entgegenwirken, ohne mit Themen der Gesundheitsvorsorge überfrachtet zu werden. Für überlappende Fragen, etwa Kontrollmöglichkeiten für DURC und die Untersuchung von Krankheitsausbrüchen unter Berücksichtigung des ganzen biologischen Risiko-Spektrums, könnte ein eigenes internationales und interdisziplinäres „Forum für Biosicherheit“ eingerichtet werden. Ein „Biosicherheits-Gipfel“ könnte auf hoher politischer Ebene initial Aufmerksamkeit für das Thema wecken und Entscheidungsträger sensibilisieren.

Die in diesem Beitrag angesprochenen Maßnahmen auszuhandeln und wirksam miteinander zu verzahnen, wäre ein zwar anspruchsvoller und voraussetzungsreicher, aber auch erfolgversprechender Weg. Er hätte den Vorteil, dass er nicht nur das politische Risikopotenzial künftiger Krankheitsausbrüche minimieren könnte, sondern auch einen der ältesten und normativ robustesten Abrüstungsverträge signifikant stärken und an aktuelle Herausforderungen anpassen würde. Die Covid19-Pandemie hat die gegenwärtigen Probleme und Defizite eindrücklich beleuchtet. Nun bleibt abzuwarten, ob die BWÜ-Mitglieder den politischen Willen zu ihrer Überwindung aufbringen.

## Footnotes

4 Gifte, die von lebenden Organismen, also z. B. Tieren, Pflanzen oder Mikroorganismen, erzeugt werden.

6 Die Konfliktlinien verlaufen dabei im Wesentlichen zwischen westlichen Staaten und der Blockfreien Bewegung (NAM) sowie Russland und China. Im Kern der zahlreichen thematischen Auseinandersetzungen stehen entgegengesetzte Ansichten dazu, ob das BWÜ durch eine Reihe einzelner Maßnahmen oder durch ein holistisches rechtlich verbindliches Instrument zu stärken wäre, sowie zum Stellenwert der entwicklungspolitischen gegenüber der abrüstungspolitischen Komponente des BWÜ.

7 Im Englischen unterscheidet man *biosafety* (Schutz vor Laborunfällen) und *biosecurity *(Schutz vor unbefugtem Zugriff).

2220 Für eine kritische Betrachtung der Vorbereitungsmission der WHO s. Shepherd et al. 2020

9 https://www.un.org/disarmament/wmd/secretary-general-mechanism/ Zugegriffen: 29. August 2020. S. auch Crowley (2018, S. 103-108).

10 Kooperation mit anderen internationalen Organisationen wie WHO oder Interpol wären ggf. wichtig. Dem UNSGM stehen im Bedarfsfall nationale Expertinnen und Experten zur Verfügung, die auch für solche Untersuchungen angefordert werden sollten. Zudem plant das VN-Abrüstungsbüro, seine eigene Expertise in diesem Bereich auszubauen (UN 2018, S. 26).

11 Informationen zu den VBM und die Formulare sind abrufbar auf der Website der ISU unter https://www.unog.ch/80256EE600585943/(httpPages)/5E2E8E6499843CCBC1257E52003ADED4?OpenDocument. Zugegriffen: 29. August 2020.
